# Health system and societal barriers for gestational diabetes mellitus (GDM) services - lessons from World Diabetes Foundation supported GDM projects

**DOI:** 10.1186/1472-698X-12-33

**Published:** 2012-12-05

**Authors:** Karoline Kragelund Nielsen, Maximilian de Courten, Anil Kapur

**Affiliations:** 1 Copenhagen School of Global Health, Department of International Health, Immunology and Microbiology, University of Copenhagen, Oester Farimagsgade 5, Bd. 9, entrance P, DK, 1353, Copenhagen K, Denmark; 2 World Diabetes Foundation, Brogaardsvej 70, DK, 2820, Gentofte, Denmark

**Keywords:** Gestational diabetes mellitus, Health system, Society, Barriers, Low- and middle-income countries

## Abstract

**Background:**

Maternal mortality and morbidity remains high in many low- and middle-income countries (LMIC). Gestational Diabetes Mellitus (GDM) represents an underestimated and unrecognised impediment to optimal maternal health in LMIC; left untreated – it also has severe consequences for the offspring. A better understanding of the barriers hindering detection and treatment of GDM is needed. Based on experiences from World Diabetes Foundation (WDF) supported GDM projects this paper seeks to investigate societal and health system barriers to such efforts.

**Methods:**

Questionnaires were filled out by 10 WDF supported GDM project partners implementing projects in eight different LMIC. In addition, interviews were conducted with the project partners. The interviews were analysed using content analysis.

**Results:**

Barriers to improving maternal health related to GDM nominated by project implementers included lack of trained health care providers - especially female doctors; high staff turnover; lack of standard protocols, consumables and equipment; financing of health services and treatment; lack of or poor referral systems, feedback mechanisms and follow-up systems; distance to health facility; perceptions of female body size and weight gain/loss in relation to pregnancy; practices related to pregnant women’s diet; societal negligence of women’s health; lack of decision-making power among women regarding their own health; stigmatisation; role of women in society and expectations that the pregnant woman move to her maternal home for delivery.

**Conclusions:**

A number of barriers within the health system and society exist. Programmes need to consider and address these barriers in order to improve GDM care and thereby maternal health in LMIC.

## Background

Although maternal mortality and morbidity have received increased attention in the last two decades it still remains a huge public health challenge in many countries. According to the World Health Organization (WHO), approximately 1000 women die from preventable causes related to pregnancy and childbirth every day with 99% of these deaths occurring in low-and middle-income countries (LMIC) [1]. Haemorrhage, hypertensive disorders, obstructed labour and infection/sepsis are among the leading global causes of maternal mortality [2]. Gestational diabetes mellitus (GDM) directly or indirectly increases the risk of all the above conditions but is rarely mentioned among the causes of maternal mortality and morbidity. Hyperglycaemia may affect <1-19% of pregnancies in LMIC [3-14] and is one of the most common medical conditions affecting pregnancy. Hyperglycaemia during pregnancy, (GDM and pre-gestational diabetes) increases the risk of maternal- and peri-natal mortality, obstructed labour, spontaneous abortion, still birth and macrosomia [15]. In countries where appropriate care for obstetrical emergencies is lacking, GDM may have particularly severe consequences for the health and well-being of the mother and child. GDM therefore represents an underestimated and unrecognised impediment to optimal maternal and neonatal health in LMIC.

Studies have shown that it is possible to reduce the risk of adverse pregnancy outcomes for women with GDM if proper management is initiated and tight glycemic control obtained [16,17]. Cost benefit of screening women for GDM particularly with the recent introduction of the International Association of Diabetes and Pregnancy Study Groups (IADPSG) guideline with the likelihood of more women being identified and requiring care is being hotly debated; these calculations are dependent on the intervention, the underlying prevalence, opportunity cost, local costing etc. Models that take into account not only the immediate pregnancy outcomes but the potential for future prevention of type 2 diabetes in the mother and offspring show cost saving or a very favourable cost effectiveness ratio [18]. Addressing GDM through early detection and proper management therefore constitutes an opportunity to improve maternal health. In the absence of an international consensus, multiple different guidelines on screening and diagnosis of GDM have existed for a long time. This may be changing with the publication of the IADPSG recommendations. While an international consensus on screening and diagnosis for GDM is welcome, it fails to take into account feasibility and applicability in low resource settings to ensure wider usage. The barriers and challenges to screening and diagnosis and the applicability of various tests have been described by us in a recent paper [19]. It is recommended that women with GDM be screened for diabetes earliest around six weeks postpartum [20-22]. After delivery most women with GDM return to normal glucose regulation, but continue to have a high risk of future diabetes, and some will be found to have overt diabetes, impaired fasting glucose or impaired glucose tolerance. To be able to identify these women and provide them all with appropriate treatment or preventive care is another opportunity and challenge. To be able to plan appropriate strategies to address these issues will require better understanding of the barriers currently hindering detection and treatment of GDM. This paper seeks to investigate societal and health system barriers hindering such efforts based on experiences gained from GDM projects supported by the World Diabetes Foundation (WDF).

## Methods

From 2002 to 2010 WDF granted support to 253 projects. In order for a project to be included in this study it had to address GDM and begun implementation of activities before March 2011. Eleven projects from eight different LMIC qualified and were included in the study. Questionnaires were sent to the project partners and the partners were asked to participate in an interview. As one project partner was implementing two of the included projects a total of 10 partners participated. All 10 responded to the questionnaire and interviews were conducted face-to-face with three partners, over the phone with six partners and via email with one partner. Participation in the study was voluntary and before the interview the purpose of it was explained to the respondents and consent to participate in the interview obtained. Permission to audio record the interview was also requested from and given by the nine project partners that were interviewed face-to-face or via telephone. Upon enquiry with the Danish Biomedical Research Ethics Committee we were assured that this study was exempt from ethical approval as it was a questionnaire and interview study without the use of human biological material.

The questionnaire was designed to obtain information about the projects, e.g. whether the project was implemented in public or private health facilities (see Additional file 1), with the intention to get a better understanding of the projects and thereby the context of the qualitative data. The interview-guide employed for the interviews was semi-structured and had mainly open-ended questions delving into barriers and challenges related to screening, diagnosis, treatment and follow-up of GDM (see Additional file 2). The list of questions and appropriate probes were drafted by KKN and AK based on literature search, previous reported challenges mentioned by WDF project partners and broad issues, e.g. barriers in the health system, which we wanted to cover in the interviews. Finally, some specific information from the questionnaire also triggered questions during the interview.

Content analysis was used to analyse the interviews, which were recorded and transcribed immediately after they were conducted, making the analysis an ongoing activity as it allowed us to be more aware of emerging themes that we could probe further during later interviews. The interviews and questionnaires were then searched for meaning units and coded by developing categories. The categories were reviewed to make sure that no categories were describing the same phenomena, and subsequently organised into core themes.

## Results

Five of the projects are implemented in India, two in Latin America and the Caribbean, two in Sub-Saharan Africa, and one in China and Sudan, respectively. Two projects, Kenya and Cuba, are implemented by either the Ministry of Health or national government institutions. The remaining projects are implemented by local NGOs, local or international research institutions, hospitals or private initiatives; however, all projects are collaborating with national or state health authorities. Six of the projects are solely implemented at public/government health centres. The other five projects are implemented at both public/government and private (including faith-based) health centres. See Tables 1 and 2 for more information about the projects.

**Table 1:** Overview of projects

**Country**	**Project title**	**Implementing partner**	**Collaborating partners**
India, Tamil Nadu	*Gestational Diabetes Mellitus – Awareness Creation, Prevention and Control in the Community*	Dr. V. Seshiah Diabetes Care and Research Institute	Department of Public Health & Preventive Medicine, Tamil Nadu; The Municipal Corporation of Chennai; Local NGOs and women’s self help groups
Cuba	*Completion of the Diabetes and Pregnancy Services Network in all the provincial capitals in Cuba*	Instituto Nacional de Endocrinología; Hospital Ginecobstétrico “Ramón Glez. Coro”.	The Maternity and Infant Program; the National Group of Obstetrics and Gynaecology; the National Group of Endocrinology; the National Committee on Diabetes and Pregnancy.
Sudan	*Gestational Diabetes Mellitus Control Project*	Sudan Fertility care Association	UNFPA Sudan Country Office; Federal Ministry of Health
Cameroon	*Improving screening, management, and outcome of gestational diabetes in urban and rural Sub-Saharan Africa*	Institute of Health and Society, University of Newcastle	Cameroon Burden of Diabetes Project; Ministry of Health
India, Tamil Nadu	*Extension of project on Gestational Diabetes Mellitus – Awareness Creation, Prevention and Control in the Community*	Dr. V. Seshiah Diabetes Care and Research Institute	Centre for Health Education and Development; Department of Public Health & Preventive Medicine, Government of Tamil Nadu
India, Karnataka	*Addressing Gestational Diabetes Mellitus in a rural and tribal Population of Mysore District, India*	Swami Vivekananda Youth Movement	Prashasa Health Consultants Pvt Ltd
Jamaica/Panama	*Strengthening Diagnosis and Treatment of Gestational Diabetes through Reinforced Maternal and Child Health Services*	International Centre for Migration, Health and Development	Ministry of Health of Panama; Ministry of Health of Jamaica
Kenya (National Programme)	*Mainstreaming Comprehensive Diabetes Care in Kenya*	Ministry of Public Health and Sanitation; The Kenya Diabetes Management and Information Centre	The Kenya Diabetes Association; the Kenya Diabetes Study Group; Kenya Diabetes Educators; the World Health Organization
India, Delhi, Jharkand, Punjab and Uttar Pradesh	*A Multi Media Approach for Awareness Generation on Gestational Diabetes and it’s Management in selected districts of India*	Jagran Pehel	Jagran Prakashan Limited; local government health departments; Indian Medical Association; Lions Club; Rotary International; Private health care facilities.
China	*China GDM centers – establishment and training dissemination*	Peking University First Hospital	Ministry of Health of China; Novo Nordisk (China)
India, Punjab	*Gestational Diabetes in Punjab*	Deep Hospital	Jagran Pehel; Sri Rama Charitable Hospital; Iqbal Hospital; Novo Nordisk; Steno Diabetes Center; Health Strategies International; Government Medical Colleges in Patiala, Amritsar and Faridkot; Municipal Corporation in Ludhiana; Department of Health and Family Welfare in Ludhiana; Copenhagen University; University of California, San Francisco.

**Table 2:** Project achievements, details and country specific maternal mortality ratio

**Country**	**Project title**	**Implemented at public/government or private (incl. faith-based) health facilities**	**No. Of women tested for GDM**	**No. Of women with GDM treated**	**No. Of health care providers trained**	**Maternal mortality ratio (per 100 000 live births)**[23]
India, Tamil Nadu	*Gestational Diabetes Mellitus – Awareness Creation, Prevention and Control in the Community*	Public	12056	1679	2550	200 [140–310]^1^
Cuba	*Completion of the Diabetes and Pregnancy Services Network in all the provincial capitals in Cuba*	Public	25066	2747	368	73 [60–87]
Sudan	*Gestational Diabetes Mellitus Control Project*	Public	7551	NA	90	730 [380–1400]
Cameroon	*Improving screening, management, and outcome of gestational diabetes in urban and rural Sub-Saharan Africa*	Both: 80% public and 20% private	12000	381	450	690 [430–1200]
India, Tamil Nadu	*Extension of project on Gestational Diabetes Mellitus – Awareness Creation, Prevention and Control in the Community*	Public	12500	1538	13860	200 [140–310] ^1^
India, Karnataka	*Addressing Gestational Diabetes Mellitus in a rural and tribal Population of Mysore District, India*	Both: 15% public and 85% private	2054	20	944	200 [140–310] ^1^
Jamaica/Panama	*Strengthening Diagnosis and Treatment of Gestational Diabetes through Reinforced Maternal and Child Health Services*	Public	NA	NA	440	110 [77–170]/92 [75–110]
Kenya (National Programme)	*Mainstreaming Comprehensive Diabetes Care in Kenya*	Both: 90% public and 10% private	NA	NA	200	360 [230–590]
India, Delhi, Jharkand, Punjab and Uttar Pradesh	*A Multi Media Approach for Awareness Generation on Gestational Diabetes and it’s Management in selected districts of India*	Both: 25% public and 75% private	NA	NA	200	200 [140–310] ^1^
China	*China GDM centers – establishment and training dissemination*	Public	26459	3230	4725	37 [23–58]
India, Punjab	*Gestational Diabetes in Punjab*	Both: 50% public and 50% private	1150	85	300	200 [140–310] ^1^

^1^Estimate for India and not for the specific state(s).

### Health system and societal barriers to GDM detection and treatment

**Figure 1:**
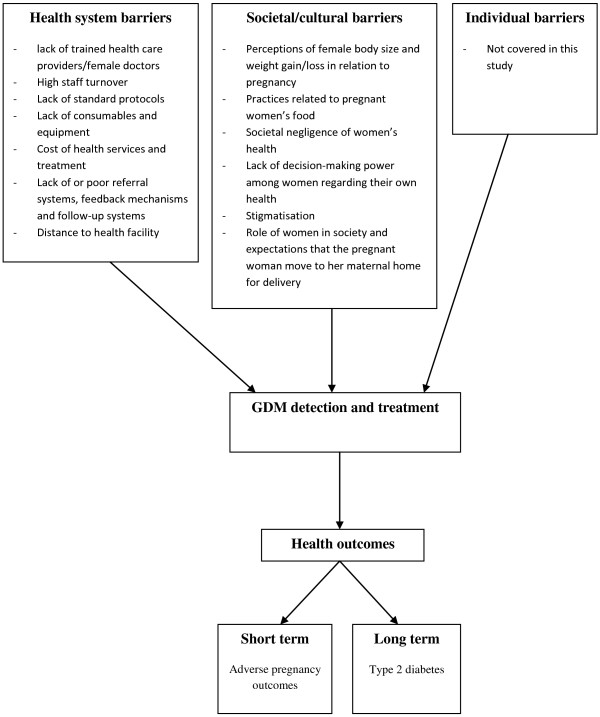
Overview of health system and societal barriers.

A number of health system and societal barriers were described by the informants. An overview of the barriers is given in Figure 1.

### Health system barriers

#### Lack of trained health care providers and high staff turnover

A main barrier within the health system was the shortage of trained health care providers. This barrier involves two dimensions: having enough health care providers to take care of the patient load and having health care providers with adequate training to provide quality care.

The first issue is the absolute critical shortage of health workers. The WHO estimates that 4.3 million more health workers are required to meet the health Millennium Development Goals (MDGs)—a global compact to reduce child mortality, improve maternal health, and combat AIDS, malaria, and other diseases by 2015 [24]. But even this alarmingly high figure significantly underestimates the global need for human resources because the WHO only accounts for shortages in 57 countries that miss the minimalist target of 2.28 doctors, nurses, and midwives per 1,000 in the population [25]. This shortage in health resources is further compounded by prioritisation based on perceptions of importance of a particular health issue and here GDM often loses the draw as respondents reported that health care planners/providers do not consider GDM important enough to prompt action as it is not part of national disease surveillance reports. When resources are limited only issues that are part of surveillance systems receive priority and get resourced.

Traditionally this is an area where the predominant diseases are the communicable diseases and much is required for malaria and HIV/AIDS. You will find that our clinics are so geared for promoting prevention of maternal to child transmission that trying now to introduce the issue of GDM takes time.

Respondent from project in Kenya

Two of the respondents from India mentioned that in their area it is not so much the number of available health care providers, but more an issue of not having enough female health care providers, especially female doctors, as many women do not want to discuss issues related to reproductive health with male doctors.

The second issue is lack of awareness and inadequate training of health care providers on GDM and the links between non-communicable diseases (NCDs) and maternal health. This is another important impediment for detection and treatment of GDM. Especially for those women who require insulin management this can be problematic as some respondents reported that health care providers in general are not sufficiently trained or confident enough in prescribing insulin.

Our health care personnel in the country are still afraid of insulin. They still try to stay far away from insulin, so when they reach a stage where they have to prescribe insulin it becomes a problem. Only few doctors would be used to prescribe insulin.

Respondent from project in Cameroon

Also lack of knowledge among health care providers about proper diet and meal plans for women with GDM were reported by respondents.

Retaining health care providers, who have received training in the area of diabetes and GDM, can be challenging as turnover of staff is quite high in some places particularly when there are limited human resources and if the learning has not been passed on to other health care providers.

It is mainly a problem with staff. Trained staff that might be posted elsewhere, so you are not sure that people you’ve trained will still be there one, two, three years later; so how to ensure that the message will go across to the whole team – that is a bit challenging.

Respondent from project in Cameroon

#### Lack of standard protocols

Another barrier mentioned is the lack of standard protocols for diagnosis and management of GDM. Consequently, some of the projects have developed such protocols themselves; yet, some of the respondents also report challenges with the development, dissemination and/or implementation of such protocols. One project for example initially intended to base their protocol on international guidelines, but discovered that many women were unable to provide the required information, making it very complicated to screen based on risk factors as recommended in the guidelines of some organisations e.g. American Diabetes Association 2010, Fifth International Workshop-Conference on GDM 2007 and National Institute for Health and Clinical Excellence 2008 [22,26,27]. These challenges have been explored in greater detail by us in an earlier paper [19].

#### Lack of consumables and equipment

Lack of test consumables and equipment were also highlighted as health system barriers for screening, detection and management of GDM. Materials needed for this include laboratory equipment, glucose solution, glucometers, equipment for monitoring foetal development as well as instruments, i.e. computers and software, for record keeping and administration. Without the necessary equipment and consumables it is next to impossible to ensure proper care and follow-up.

Field staff can be trained, field staff can be motivated, field staff can be encouraged and become willing to do it, as long as they have the way and tools to do it. It is no good asking field staff to do something that they don’t have the equipment to do, the time to do or the knowledge to do.

Respondent from project in Jamaica and Panama

#### Financing of health services and treatment

Another issue is the lack of health financing for screening and treatment, i.e. when the patient is obliged to pay a fee for screening and/or treatment services and consumables. However low the cost, paying out of pocket is a barrier to access care and adhere to treatment for many women with GDM in LMIC. Not only is the cost of medication a barrier but even the cost of following the recommended diet can be challenging for many.

The other obstacle will always be whether changing a diet is economically feasible. I think we should really pay a lot of attention to that when we are dealing with GDM.

Respondent from project in Jamaica and Panama

In addition, one of the respondents from India noted that although services within the government health care system may be offered free of charge or at subsidised rates, the lack of trained health care providers in reality leaves some women with GDM with no choice other than to seek care at private health facilities with considerably higher costs and this option is not possible for women with GDM belonging to the poorer segments of society. Thus, the cost of the treatment as well as fee for services, i.e. consultations and tests, in some contexts constitutes a barrier for proper treatment; health financing mechanisms therefore not only need to address access and costs, but also quality and comprehensiveness of the programmes.

#### Lack of referral systems, feedback mechanisms and follow-up systems

Another issue mentioned is lack of functioning referral systems and feedback-mechanisms especially in cases where treatment is not offered at the primary health care level but at more specialised clinics. Women, who are referred to other clinics for care, may be lost between the referring and the reference health facility, if neither of the two follows up on whether she actually attends the other institution. Similarly, when there is no feedback, the health care providers at the primary health care level either often do not refer patients or if unwilling to deal with GDM, may refer all cases to specialist centres. Moreover, even when women are treated at the same clinic where screening and diagnosis take place, continuous follow-up before, during and after delivery poses a challenge when no follow-up system is in place. This is particularly true with regard to post-partum follow-up and care, when the woman no longer has diabetes (but both the mother and child carry a very high risk of future diabetes) and therefore is neither seen by the obstetrician nor the diabetes specialist and is considered lost in the system. Nonetheless, both the mother and child may be visiting the same health facility for the well-baby clinic or immunization programme but the system fails to identify them to provide continued counselling because of lack of communication between different departments.

#### Distance to health facility

Transportation to the health centre, both in terms of the cost and the distance can also constitute a barrier to early detection, diagnosis and treatment of GDM according to respondents. The latter in particular can be affected if the woman lives far from the health facility as the woman then is required to attend the facility regularly for monitoring. Travelling long distances under difficult travel conditions during advanced pregnancy has many challenges and often requires that the women be accompanied by an escort further adding to the cost and feasibility. Therefore it is not surprising that women in most LMIC particularly from rural areas reportedly have much fewer antenatal visits.

### Societal barriers

#### Perceptions of female body size and weight gain/loss in relation to pregnancy

In some countries societal or cultural issues can hamper treatment of GDM. Some respondents reported on local perceptions of the desirable body size and shape of women, not being conducive to motivating them to improve eating habits and lose weight.

In Jamaica for example the ideal body size is big… So when you are dealing with people who have a body image which means that being large and heavy is quite acceptable and maybe even attractive then it is very difficult to try and get people to change their diet.

Respondent from project in Jamaica and Panama

Moreover, the issue of eating habits or losing weight during pregnancy may be particularly sensitive in some areas.

People are not comfortable about the idea of not gaining enough weight during pregnancy. They just feel it means you are sick, that you have some sort of disease. So they would want to put on some weight during pregnancy, and when I say ‘some weight’ the understanding of ‘putting on some weight’ can vary a lot. So the idea of putting on weight during pregnancy is something important to them. In urban areas it won’t be the same, but in semi-urban and rural areas they are not even expected to lose weight after giving birth so they are sometimes overfed by the family after delivery just to keep as big as they were during pregnancy.

Respondent from project in Cameroon

Notions like these are not only problematic for treatment of GDM, but also for the postpartum prevention of future onset of type 2 diabetes.

#### Practices related to pregnant women’s diet

Other aspects related to diet were also brought out by the respondents. For instance, one respondent from India noted that it is customary to encourage pregnant women to eat sweets and certain calorie dense, high fat snacks in order for them to have enough energy, and people bring such food as gifts when they visit. Thus, being on diet where such things are banned can be a damper on the celebrations of the pregnancy and child birth within the family and raise issues about the health of the young woman thereby curbing her motivation to eat healthy.

Moreover, it was stated that it would not always be considered appropriate for women in India to have special low-calorie food for herself as she is expected to eat the same as the rest of the family and not attract much attention to herself and her needs.

#### Societal negligence of women’s health

Moreover, cultural notions about women and the importance of their health also emerged as a barrier. Some respondents explained that sometimes the woman’s family may not consider her health to be important enough to spend the extra money on healthy foods or treatment. This may especially be the case after delivery as the health of the woman is no longer seen as influencing the health of the baby.

The health of women in India is the most neglected, under-looked and deficient system of the whole country. People cannot be bothered. They are just not bothered about the health of women whether it is diabetes or anything else.

Respondent from project in Punjab, India

#### Lack of decision-making power among women regarding their own health

In many cultures the woman herself does not make the decisions even those concerning her own health - those decisions are generally made by her husband and/or in-laws, and if they make the decision that she should not attend antenatal care or not have a specific test performed it is very difficult for her to demand the test.

Whether a woman should go for antenatal check-up or not is a decision taken by her husband, if she goes there and she finds that there is some problem, what kind of treatment, which doctors she should consult etc. – all these decisions are being taken by the male counterpart.

Respondent from project in multiple states of India

#### Fear of stigmatisation

Another impediment noted by some of the respondents is that it can be highly stigmatising for a woman to be diagnosed with GDM and the consequences of this for her can be intimidating.

That fear inside her that everything will go wrong in her life. Even if it is a risk or recommendation from a doctor, a call from a doctor that ‘you are at risk of getting diabetes’ or ‘the child will get affected with some borderline hyperglycaemia’ would probably ruin her family life - her husband would not look upon her nicely or her mother-in-law will always be sarcastic in her remarks.

Respondent from project in Punjab, India

Therefore, some women refuse the test simply because they fear the consequences of its result. Yet, even among women who are diagnosed with GDM postpartum testing for overt diabetes is a challenge because of such fears as a diagnosis of overt diabetes can be devastating for her in financial, emotional and social terms.

#### Role of women in society

A number of more practical aspects were also mentioned as barriers to GDM detection, diagnosis and treatment. Many of these are related to the woman’s role in society - having to take care of the children and doing other chores related to the household. Being too busy to have time to attend antenatal care and GDM testing was therefore cited as another barrier to ensure early detection of GDM. The issue revolves around both the time consumed on the test and the time spent on transport to and from the health centre. This is an even bigger issue in terms of long term follow-up of women with GDM to address future prevention of diabetes. Even if one tries to establish follow-up mechanism through the well-baby or vaccination programme it may not work because the child may be brought to the clinic by somebody else – a grandparent because the women is required to deal with the household chores. Creating outreach home visit based services are therefore very important in these contexts.

#### Expectations that the pregnant woman move to her maternal home for delivery

Some respondents noted that in their area, women tend to move to their maternal home before delivery, adding a further barrier to care delivery and follow-up as the health care provider in the new area may not have the full records, may not be well versed with the case or may not have the training to deal with GDM.

## Discussion

In this study a number of barriers to improving maternal health related to GDM were identified, including lack of trained health care providers - especially female doctors; staff turnover and lack of standard protocols, consumables and equipment; financing of health services and treatment; lack of or poor referral systems, feedback mechanisms and follow-up systems; distance to health facility; perceptions of female body size and weight gain/loss in relation to pregnancy; practices related to pregnant women’s diet; societal negligence of women’s health; lack of decision-making power among women regarding their own health; stigmatisation; role of women in society and expectations that the pregnant woman move to her maternal home for delivery.

According to our knowledge only a few studies have previously investigated barriers to management or postpartum follow-up of women with diabetes during pregnancy [28-32], and none of these are from LMIC. Although these studies were conducted in a setting very different from our study there are certain similarities between the findings of these studies and ours. Hence, Bennet et al., Collier et al., Mersereau et al. and Razee et al. reported lack of concern about women’s health – either because they feel healthy or because they have less time for self-care due to the demands of the baby or other responsibilities – as a barrier to GDM management or postpartum follow-up [28-31]. Fear of being diagnosed with overt diabetes was also identified by Bennet et al. as a barrier, although the reason behind this fear was not grounded in fear of stigmatisation, but more the prospect of having to follow a strict diet and regularly having to attend medical check-ups [28]. Collier et al. also identified cost of health services, diabetic supplies and healthy foods as barriers [29]. Finally, difficulties in accessing care and cultural issues impeding healthy diet and physical exercise were also identified by Razee et al., Mersereau et al. and Collier et al. as barriers to GDM management [29-31].

Considering that WHO in the 2006 World Health Report concluded that there is a global shortage of almost 4.3 million doctors, nurses, midwives and support workers [24] it is not surprising that turnover and lack of trained health care providers is mentioned as a barrier for GDM services. Seven of the projects included in this study are implemented in countries where WHO assess there is a critical shortage of health service providers [24]. India is one of these countries and as only around 10% of doctors in South-East Asia are women [24] it is not surprising that respondents from India noted lack of female doctors as a particular problem.

However, as indicated by the respondents it is not only a problem of numbers it is also a problem of skills and training. Lack of knowledge has also been found to be a problem for the management of type 2 diabetes in LMIC [33,34]. Thus, to ensure that women with GDM receive proper treatment, training of health care providers need to be initiated or scaled up. Lack of standard protocols on GDM diagnosis and management was also identified as a barrier to early detection and proper management of GDM. The lack of such protocols may reflect the limited attention that GDM has received in many LMIC, the lack of international consensus on the diagnostic criteria for GDM as well as existing protocols in their current form not being feasible to implement in many LMIC [19].

In addition, findings from this study also illustrate that health services and systems are disorganised and inadequately financed and can work as barriers for achieving specific health-related outcomes, in this case GDM detection and treatment. This is far from new, but health system planners and policy-makers should take these structures and aspects into account when initiating GDM services.

A substantial number of the barriers are societal or culture-related e.g. expectations that the woman transfers to her maternal home to deliver. While their relevance may vary, such barriers remain important according to our findings. Yet, they are largely beyond the realm of the health sector and therefore have to be addressed outside it through awareness and policies. Issues related to women’s role in society and how much emphasis is given to their health and well-being seems to be of particular concern and the findings from this study indicate that much still remains to be done to ensure women’s empowerment including the right to control all aspects of their health as stated in the Beijing Declaration adopted at the UN Fourth World Conference on Women in 1995 [35].

Finally, in this study all participants were WDF project partners and many of them are also practicing health care providers. In order to further illuminate the issue it would be important to undertake studies where women and their families are interviewed about barriers and facilitators for GDM services. Such a study should also focus on barriers within the control of the individual in addition to health system and societal/cultural barriers.

## Conclusion

In this paper we examined barriers to GDM detection and treatment in health systems and society. In order to provide effective GDM services to improve maternal health in LMIC, programmes have to consider and address these barriers.

## Competing interest

AK is currently employed by the World Diabetes Foundation which provided financial support to the projects included in this article. KKN was employed by the World Diabetes Foundation when the study was conducted. MdC has no conflict of interests to declare.

## Authors’ contributions

KKN participated in the conception and design of the study, conducted the interviews, participated in the analysis and drafted the manuscript. MdC contributed to the interpretation of data and the drafting of the manuscript. AK participated in the conception and design of the study, contributed to the analysis and interpretation of data and the drafting of the manuscript. All authors read and approved the final manuscript.

## Pre-publication history

The pre-publication history for this paper can be accessed here:

http://www.biomedcentral.com/1472-698X/12/33/prepub

## Supplementary Material

Additional file 1 Questionnaire. Click here for file

Additional file 2 Interview guide. Click here for file
