# Supplementary material for: Health system and societal barriers for gestational diabetes mellitus (GDM) services - lessons from World Diabetes Foundation supported GDM projects
Source: BMC Int Health Hum Rights. 2012 Dec 5;12:33. doi: 10.1186/1472-698X-12-33 (PMC3552986; doi:10.1186/1472-698X-12-33)
Supplement: Additional file 1 — Questionnaire. [file 1472-698X-12-33-S1.docx]

Appendix 1 Questionnaire

1. Are your project population living in an urban, semi-urban or rural area? (Please mark with an “X”. Several marks can be made)

- Urban

- Semi-urban

- Rural

- Other

2. How far are you in the implementation phase? (Please mark with an “X”. Only one mark can be made)?

- In the beginning

- About 1/4 in

- Mid-way

- About ¾ in

- Almost at the end

- Project is completed

- Other (please specify)

3. In addition to GDM do you address maternal malnutrition and general maternal health in your project? (Please mark with an “X”. Only one mark can be made)

- Yes (please specify how) ____________________________

- No

- Other (please specify)_______________________________

4. Is your project being implemented in public or private health facilities? (Please mark with an “X”. Only one mark can be made)

- Public (government)

- Private (both non-profit and for profit)

- Both.

- Other

4a. If your project implementation includes both public and private health facilities, please specify the proportion of each (e.g. 20% public and 80% private)

______________________________________

5. Which activities does your project include? (Please mark the activities with an “X”. Several marks can be made):

- Screening
- Treatment
- Patient education
- Awareness raising among general population
- Advocacy
- Training or building capacity among health care providers
- Establishing or strengthening clinics through procurement of equipment
- Developing and/or disseminating protocols/guidelines
- Establishing or strengthening referral system
- Establishing or strengthening health information system
- Assessment of GDM prevalence
- Other (kindly specify what)

The following questions relate to some of the above mentioned activities. If your project is not conducting the mentioned activity please skip the question.

*Screening*

6a. *Screening*. What diagnostic test do you use when screening and diagnosing GDM?

6b. *Screening*. What cut-off values do you use to diagnose GDM?

6c. *Screening*. Do you screen all pregnant women for GDM (universal screening) or only pregnant women with risk factors for GDM (selective screening)?

6ca. *Screening*. If only women with risk factors are screened for GDM, please specify which risk factors you use to assess whom to screen.

6d. *Screening*. When are the women screened for GDM? (Please mark with an “X”. Only one mark can be made)

- At first antenatal visit regardless of gestational week
- At screening camps regardless of gestational week
- Before 24^th^ week of gestation
- 24-28 weeks of gestation
- After 28^th^ week of gestation
- Other (please specify)

6e. Do you screen women 4 to 6 weeks post delivery to confirm that women do not have diabetes?

*Treatment and management protocols*

7a. *Treatment and management protocols*. What treatment/management are you recommending to women with GDM in your project?

7b. *Treatment and management protocols*. What blood glucose levels are you aiming for in the treatment/management of women with GDM?

7c. *Treatment and management protocols*. Kindly, describe in brief how women with GDM are followed before, during and after delivery in your project.

7d. *Treatment and management protocols.* Please specify the proportion of women with GDM in your project population who are treated with insulin.

7e. *Treatment and management protocols. Is* treatment/management of GDM offered free of charge?

(Please mark with an “X”. Only one mark can be made)

- Yes, free of charge for all women with GDM
- No, all women with GDM pays the same fee
- The poorest are not charged for treatment. Others are charged a fee.
- All are charged, but the poorest are charged less.
- Other (please specify)

*Patient education*

8b: *Patient education* Who is offered patient education related to GDM?

(Please mark with an “X”. Only one mark can be made)

- Only women diagnosed with GDM
- Women diagnosed with GDM and their family
- All pregnant women attending antenatal care
- Other (please specify)

8b: *Patient education.* What is the key message(s) that is disseminated in patient education related to GDM in your project?

*Awareness raising among the general population*

9a. *Awareness raising among the general population.* Kindly, specify who your key target audience(s) is?

______________________________________________

9B. *Awareness raising among the general population.* What is the key message(s) that is disseminated in your awareness raising activities related to GDM?

9c. *Awareness raising among the general population.* What communication channels do you employ when raising awareness related to GDM?

9d. *Awareness raising among the general population.* In your experience, what has been the most effective means of awareness raising? – Please elaborate why.

*Advocacy*

10a. *Advocacy.* Please, list the advocacy activities that you have been conducting related to GDM?

10b. *Advocacy.* Who is the target group for your advocacy activities?

10c. *Advocacy.* What is the objective of your advocacy activities?

10d. *Advocacy.* If relevant, please list outcomes from your advocacy activities (e.g. changes in policy, increased support from village leaders etc).

*Assessment of GDM Prevalence*

11a. *Assessment of GDM Prevalence.* If you have analysed your results, kindly, specify what the GDM prevalence is in your project area.
