# Supplementary material for: Health system and societal barriers for gestational diabetes mellitus (GDM) services - lessons from World Diabetes Foundation supported GDM projects
Source: BMC Int Health Hum Rights. 2012 Dec 5;12:33. doi: 10.1186/1472-698X-12-33 (PMC3552986; doi:10.1186/1472-698X-12-33)
Supplement: Additional file 2 — Interview guide. [file 1472-698X-12-33-S2.docx]

**Appendix 2 Interviewguide**

1. In your opinion, what are the main barriers to ensuring early and proper diagnosis and management of women with GDM?
2. What are the main barriers from the woman’s perspective?
3. Are there any social or economic barriers that might prevent women from getting screened and treated for GDM?
4. Are there any cultural or religious barriers that might prevent women from getting screened and treated for GDM?
5. Many projects have noted difficulties with ensuring follow up of women diagnosed with GDM before, during and after delivery, what are your experiences with this?
6. Why are the women lost to follow up?
7. What do you think could be done to address this loss to follow up?
8. From a health system point of view, what are the main barriers for ensuring early and proper diagnosis and management of women with GDM?
9. I can see from your response to the questionnaire/project reports that you are using the XXXX method for screening for GDM. The diagnostic criteria for GDM have been an issue of debate, why did you decide to use this method?
10. Why did you choose universal or risk factor based screening?
11. In your experience, how has your efforts to address GDM been received by the local health care providers?
12. How are your own experiences regarding support from authorities/ decision-makers when it comes to addressing GDM?
13. In your opinion, what are the major barriers to ensuring support from authorities/ decision-makers for screening and management of women with GDM?
14. How do you think such support can be ensured?
15. Overall, what are the key lessons that you have learned so far from implementing this GDM project?
16. If you should point at one thing which would contribute the most to improving GDM screening and treatment what would it be?

What could:

- Your own organisation do?

- Similar organisations do?

- Local/national decision/policy-makers do?

- WDF do?
